# Supplementary material for: Leaving the health workforce during the COVID-19 pandemic: A cross-sectional study among Filipino healthcare workers
Source: PLOS Glob Public Health. 2025 Oct 15;5(10):e0004861. doi: 10.1371/journal.pgph.0004861 (PMC12527175; doi:10.1371/journal.pgph.0004861)
Supplement: S1 Text — (DOCX) [file pgph.0004861.s002.docx]

**Survey Questionnaire**

**Section 1: Inclusion**

Are you a Filipino health care worker?*

- Yes
- No

What is your occupation?*

- Medical Technologist
- Nurse
- Occupational Therapist
- Physical Therapist
- Physician/Doctor
- Radiologic Technologist
- Speech Pathologist
- Others: (please specify)

Did you work in the hospital during the COVID-19 pandemic?*

- Yes
- No

Did you leave your designation in the hospital anytime during the COVID-19 pandemic (starting March 11, 2020)?*

- Yes
- No

Indicate the month and year when you left your designation in the hospital. Please give your best estimate of your last day at work*

How long have you been practicing as a healthcare worker (*number of years)?**

What were the circumstances regarding your termination from work?*

- I resigned before the end of my contract
- My contract expired and I did not renew anymore
- I went AWOL (absence without leave)
- I was relieved from work by my employer (e.g., because of budget cuts, etc)
- Others: (please specify)

Did you intend to leave your designation even prior to the pandemic?*

- Yes
- No
- Prefer not to say

If yes, what was your reason for planning to leave prior to the pandemic?

- I wanted to retire early
- I planned to migrate and work abroad
- I was actively seeking new employment in the Philippines
- I wanted to study further
- Others, please specify:

Did your family convince you to stop working in the hospital?

- Yes
- No
- Prefer not to say

What is your current occupation or source of livelihood?

**Section 2: Demographic information**

What is your gender?*

- Male
- Female
- Non-binary (identifies as part of the LGBTQIA+)
- Prefer not to say

What is your age?*

What is your highest educational attainment?*

- High school
- Vocational degree
- Bachelor's Degree
- Postgraduate Degree (MD, DDM, Masters, Doctorate, etc.)

Which of the following describes you?*

- Single, never married
- Married or in a long-term relationship
- Separated
- Widowed

Do you have any children?*

- Yes
- No

Are you the primary breadwinner in your household (household: group of people living in the same house and/or sharing resources like food, water, etc. and may include your nuclear or extended family)? *

- Yes
- No

Is there anyone else contributing to your household income? *

- Yes
- No

Do you have any of the following health conditions? Please check all that apply.*

- I don’t have any health condition/s
- Hypertension
- Diabetes mellitus
- Asthma
- Obesity
- Cancer
- Tuberculosis
- Others: (please specify)

**Section 3: Work information**

Where do you practice as a healthcare worker?*

- NCR: National Capital Region
- CAR: Cordillera Administrative Region
- Region I: Ilocos Region
- Region II: Cagayan Valley
- Region III: Central Luzon
- Region IV-A: Calabarzon
- Region IV-B: Mimaropa: Southwestern Tagalog Region
- Region V: Bicol Region
- Region VI: Western Visayas
- Region VII: Central Visayas
- Region VIII: Eastern Visayas
- Region IX: Zamboanga Peninsula
- Region X: Northern Mindanao
- Region XI: Davao Region
- Region XII: Soccsksargen
- Region XIII: Caraga
- BARMM: Bangsamoro

Did you provide direct patient care to confirmed, probable, or suspected COVID-19 patients? *

- Yes
- No

Which of the following best describes your workplace? *

- Level 1 Hospital (primary or district hospital)
- Level 2 Hospital (secondary or provincial hospital)
- Level 3 Hospital (tertiary, regional, national, university/teaching hospital)

Which of the following best classifies your workplace?*
(If your hospital is a government hospital but with private wards/pay floors, please select Government)

- Government
- Private

Where did you spend most of your time in the workplace?*

- Outpatient clinic
- Emergency department
- Ward
- ICU
- Operating room
- Laboratory
- Office
- Other

How long were your work hours per day, excluding break and commute?*

- ≤ 4 hours
- 5-8 hours
- 9-12 hours
- 13-16 hours
- 16-24 hours

In a given shift, estimate the number of patients you directly cared for:

What was your estimated NET monthly salary (amount after taxes) as a healthcare worker when you were working in the hospital?

Please select the reasons why you quit your work in the hospital (check all that apply):

- Weakening of benefits (e.g. retirement contributions/pensions, health insurance)
- Work overload/burnout
- Workplace environment
- Lack of acknowledgement/recognition
- Lack of satisfaction with your supervisor
- Lack of opportunities for advancement
- Lack of flexibility (flex hours/telework)
- Lack of Job satisfaction
- Lack of support at work
- Lack of training/continuing education
- Leadership or administration change
- Other opportunities outside agency
- Fear of getting COVID-19
- Insufficient pay/salary
- Retirement
- Stress
- Other: _______

What interventions, when implemented, will convince you to return to work or encourage you to stay as a hospital healthcare worker during the pandemic?*

At what age do you plan to retire/leave the healthcare profession?

**Culture of Care Barometer (Rafferty, et al., 2017), adapted and edited**

Think of your previous workplace (i.e. hospital). Please indicate the extent you agree with each of the following statements by ticking one box on each row. This tool is intended to encourage self-reflection, so take your time to consider each statement.*

|  | Strongly disagree | Disagree | Neither | Agree | Strongly agree |
| --- | --- | --- | --- | --- | --- |
| I have the resources I need to do a good job |  |  |  |  |  |
| I feel respected by my co-workers |  |  |  |  |  |
| I have sufficient time to do my job well |  |  |  |  |  |
| I am proud to work in this hospital |  |  |  |  |  |
| My line manager (i.e., supervisor) treats me with respect |  |  |  |  |  |
| The hospital values the service we provide |  |  |  |  |  |
| I would recommend this hospital as a good place to work |  |  |  |  |  |
| I feel well supported by my line manager |  |  |  |  |  |
| I am able to influence the way things are done in my team |  |  |  |  |  |
| I feel part of a well-managed team |  |  |  |  |  |
| I know who my line manager is |  |  |  |  |  |
| Unacceptable behavior is consistently tackled |  |  |  |  |  |
| There is strong leadership at the highest level in the hospital |  |  |  |  |  |
| When things get difficult, I can rely on my colleagues |  |  |  |  |  |
| Hospital managers know how things really are |  |  |  |  |  |
| I feel able to ask for help when I need it |  |  |  |  |  |
| I know exactly what is expected of me in my job |  |  |  |  |  |
| I feel supported to develop my potential |  |  |  |  |  |
| A positive culture is visible where I work |  |  |  |  |  |
| The people I work with are friendly |  |  |  |  |  |
| My line manager gives me constructive feedback |  |  |  |  |  |
| Staff successes are celebrated by the administration |  |  |  |  |  |
| The administration listens to staff views |  |  |  |  |  |
| I get the training and development I need |  |  |  |  |  |
| I am able to influence how things are done in the hospital |  |  |  |  |  |
| The hospital has a positive culture |  |  |  |  |  |
| I am kept well informed about what is going on in our team |  |  |  |  |  |
| I have positive role models where I work |  |  |  |  |  |
| I feel well informed about what is happening in the hospital |  |  |  |  |  |
| My concerns are taken seriously by my line manager |  |  |  |  |  |

**Section 4: Worry and concern among healthcare workers regarding the COVID-19 pandemic (adapted and edited from of Sahashi, et al., 2021)**

| How worried are you about COVID-19 pandemic* | I’m not worried at all | I am not worried | I am neither | I am worried | I am terribly worried |
| --- | --- | --- | --- | --- | --- |

Check off the items that cause you concern. (multiple responses are possible)*

- The dangers of the disease itself
- The risk of infection in family members and other relatives
- Isolation from family and the social environment
- The impact of becoming infected on my family, work, and society

|  | Strongly disagree | Disagree | Neither | Agree | Strongly agree |
| --- | --- | --- | --- | --- | --- |
| The wards and departments to which you belonged were well prepared for the COVID-19 pandemic |  |  |  |  |  |
| The risk of being infected with COVID-19 in the workplace is very high |  |  |  |  |  |
| The recommended measures are very effective |  |  |  |  |  |
| The required personal protective equipment (masks, globes, protective equipment) is not available |  |  |  |  |  |
| Being infected with COVID-19 will have major consequences to your health |  |  |  |  |  |
| COVID-19 is difficult to treat |  |  |  |  |  |
| Psychological support services for your concerns about the COVID-19 pandemic are important |  |  |  |  |  |

Have you already been infected with COVID-19?*

- Yes
- No
- Prefer not to say

Have your co-workers been infected with COVID-19?*

- Yes
- No
- Prefer not to say

Has any member of your immediate family (e.g., spouse, children, parents, siblings) been infected with COVID-19?*

- Yes
- No
- Prefer not to say

[END OF SURVEY]
