# Supplementary material for: Leaving the health workforce during the COVID-19 pandemic: A cross-sectional study among Filipino healthcare workers
Source: PLOS Glob Public Health. 2025 Oct 15;5(10):e0004861. doi: 10.1371/journal.pgph.0004861 (PMC12527175; doi:10.1371/journal.pgph.0004861)
Supplement: S1 List — (DOCX) [file pgph.0004861.s003.docx]

**List of Organizations**

| **Organization** | **Contact used** |
| --- | --- |
| Philippine Academy of Occupational Therapists | <https://www.facebook.com/PAOTInc>  <https://twitter.com/PAOTinc> paot.org@gmail.com |
| Philippine Physical Therapy Association | <https://www.facebook.com/pptainc>  <https://twitter.com/phil_pt_a>  pptaboard@gmail.com |
| Philippine Association of Speech Language Pathologists | <https://www.facebook.com/pasp.org.ph>  <https://twitter.com/upcasp> <https://twitter.com/pasp_org> pasp.secretary@gmail.com |
| The Philippine Association of Medical Technologists, Inc. | <https://www.facebook.com/pametofficial>  [pametphilippines@yahoo.com.ph](mailto:pametphilippines@yahoo.com.ph) |
| The Philippine Association of Radiologic Technologists, Inc. | <https://www.facebook.com/PARTinc62nd/> |
| Filipino Nurses United | filipino_nurses2015@yahoo.com |
| Philippine Nurses Association | philippinenursesassociation@yahoo.com.ph |
| Philippine College of Physicians | <https://www.facebook.com/pcpofficialpage>  <https://twitter.com/pcp1953> secretariat@pcp.org.ph |
| Alliance of Health Workers - AHW National | [ahwphil@yahoo.com](mailto:ahwphil@yahoo.com) |
| NKTI Employees Association | <https://www.facebook.com/NKTI-Employees-Association-AHW-2363226140386791> |
| Paranaque Health Workers Association | <https://www.facebook.com/ParanaqueHealthWorkersAssociation> |
| AKSYON Health Workers | aksyonhw@gmail.com |
| Pangkalusugang Lingkod Bayan | plb.upm@gmail.com |
| Philippine Medical Association | philmedas@yahoo.com |
| Philippine Hospital Association | philhospitalassn@gmail.com |
| Philippine College of Hospital Administrators | pchainc@yahoo.com |
| Private Hospitals Association Philippines | [eulama@yahoo.co](mailto:eulama@yahoo.co)  <https://www.phap-ph.org/Contacts/message> |
| All U.P. Workers Union | [aupwunational@gmail.com](mailto:aupwunational@gmail.com) |
| Healthcare Professionals Alliance for COVID-19 | secretariat@hpaac.org.ph  <http://hpaac.org.ph/contact> |
| AMSA-Philippines | evp.amsaphil@gmail.com |
