# Supplementary material for: Leaving the health workforce during the COVID-19 pandemic: A cross-sectional study among Filipino healthcare workers
Source: PLOS Glob Public Health. 2025 Oct 15;5(10):e0004861. doi: 10.1371/journal.pgph.0004861 (PMC12527175; doi:10.1371/journal.pgph.0004861)
Supplement: Attachment — Submitted filename: Response to reviewers.docx [file pgph.0004861.s009.docx]

June 10, 2025

Padmasayee Papineni, MD

Academic Editor

*PLOS Global Public Health*

Dear Dr. Papineni:

Thank you for reviewing and providing valuable feedback on our manuscript.

PGPH-D-24-02821

**Title:** Leaving the health workforce during the COVID-19 pandemic: A cross-sectional study among Filipino healthcare workers

**Authors:** Christl Jan S. Tiu, Nicole Rose I. Alberto, Maria Beatriz C. Baron, Michael Vincent V. Mercado, Ronnie E. Baticulon, MD

As we aim to provide a paper at par with your journal’s expectations, our team has addressed the points raised by the reviewers to strengthen the quality of our manuscript through the following:

|  | Reviewer comment: | Action taken: |
| --- | --- | --- |
| 1 | “One notable limitation of the study is the disproportionate representation of respondents from the National Capital Region (NCR), which accounts for a significant portion of the sample. Given that the NCR is not proportionate to the overall population of healthcare workers in the Philippines, this geographic imbalance may limit the generalisability of the findings to healthcare workers in other regions, who may face different challenges and circumstances. For instance, healthcare workers in rural areas might have distinct experiences related to resources, infrastructure, and access to support systems compared to their counterparts in urban hospitals like the NCR. To strengthen the validity of the study, it would be beneficial to address this sampling bias by including a more regionally representative cohort of participants or by discussing how this limitation might have influenced the results.” | Thank you for this comment. We recognize the limitation of the disproportionate sampling of healthcare workers. Given the limitation of being unable to add more to the participants to the study, we added an acknowledgement and explanation in the limitation section and included a proposal for future studies to explore a more regionally representative cohort of participants.  Manuscript with track changes, Page 15, line 274 to 279 |
| 2 | “It would be helpful if the author examined how the cohort is distributed between healthcare workers in the public and private sectors. In the Philippines, there is a substantial salary gap between these sectors, which could influence attrition rates. Exploring whether attrition is higher among healthcare workers in public hospitals compared to private hospitals could provide valuable insights and may further contextualise the findings. Including this analysis or acknowledging its absence as a limitation would strengthen the study.” | Thank you for this comment. While the data gathered are proportionate in terms of the total healthcare professionals from the public and private sector (46% and 54%), there is unequal representation of public and private professionals across each healthcare worker occupation. As such, the data may be insufficient to employ formal statistical analysis on factors for each sector. Salary differences for selected occupations (nurses and physicians) were included in the main revised manuscript, accompanied by an explanation in the limitation section as well as a supplementary table of the data in S2_tables supplementary document.  Further, we used “government employment” in the manuscript instead of “public” in page 9, line 146 to 147 to describe the difference. To address and prevent confusion for future readers, we revised the wording into “public sector” in the revised manuscript and “public hospital” in the supplementary tables instead.  Manuscript with track changes, Page 9   - line 147 to 149 - line 158 to 161   Manuscript with track changes, Page 15, line 279 to 283  S2_tables with track changes, page 1, Supplementary Table 1., Column 1 Ownership, Column 2 Subcategory - replaced “Government” to “public”  S2_tables with track changes, page 2, Supplementary Table 3. |
| 3 | “1. Table 1 – Sociodemographic characteristics of survey respondents. Please review column 1-category on ‘Years practicing as healthcare worker’-subcategory under column 2, range 16 to 20 is missing.” | Thank you for pointing this out. Our team initially decided to remove the range 16 to 20 as it had zero (0) responses. To address this, we have placed the range 16 to 20 accordingly to prevent confusion among readers.  Manuscript with track changes, Page 8, Table 1, Column 1 - Years practicing as healthcare worker, Column 2 - added range “16 to 20”. |

I hope the concerns raised have been addressed in line with the standards your esteemed journal upholds. Should there be any additional requirements or clarifications needed, please do not hesitate to inform us. We are grateful for the opportunity to submit our manuscript and remain hopeful for its favorable consideration.

Thank you very much for your time!

Sincerely,

Christl Jan S. Tiu, M.D. *College of Medicine, University of the Philippines Manila*

Ronnie E. Baticulon, M.D. *College of Medicine, University of the Philippines Manila*
